# Effects of *Salmonella enterica* serovar Enteritidis infection on egg production and the immune response of the laying duck *Anas platyrhynchos*

**DOI:** 10.7717/peerj.6359

**Published:** 2019-01-25

**Authors:** Yu Zhang, Yang Chen, Tiantian Gu, Qi Xu, Guoqiang Zhu, Guohong Chen

**Affiliations:** 1 Joint International Research Laboratory of Agriculture & Agri-Product Safety of the Ministry of Education, Yangzhou University, Yangzhou, China; 2 College of Veterinary Medicine, Yangzhou University, Yangzhou, China

**Keywords:** Duck, *Salmonella enterica* serovar enteritidis, Reproductive tract, Bacterial distribution, Immune gene

## Abstract

Persistent colonization of the avian reproductive tract by *Salmonella enterica* serovar Enteritidis (SE) negatively affects egg production and contaminates the egg. The immune function of the ovary and oviduct is essential for protection from infection and for the production of wholesome eggs. However, the immune response of laying ducks during SE infection is not well-understood. In this study, ducks (*Anas platyrhynchos*) were infected with SE and were systematically monitored for fecal shedding during a 13-week period. We also assessed bacterial distribution in the reproductive tract and classified infected ducks as resistant or susceptible based on the presence of tissue lesions and on SE isolation from fecal samples. We found that infected animals had persistent, but intermittent, bacterial shedding that resulted in the induction of carrier ducks. Laying rate and egg quality were also decreased after SE infection (*P* < 0.05). SE readily colonized the stroma, small follicle, isthmus, and vagina in the reproductive tracts of susceptible ducks. Immunoglobulin (IgA, IgG, IgM) levels were higher in susceptible ducks compared with resistant birds (*P* < 0.05); T-lymphocyte subpopulations (CD3^+^, CD4^+^, CD8^+^) displayed the opposite trend. qRT-PCR analysis was used to examine expression profiles of immune response genes in the reproductive tract of infected ducks. The analysis revealed that immune genes, including toll-like receptors (TLR2, TLR4-5, TLR15, TLR21), NOD-like receptors (NOD1, NLRX1, NLRP12), avian β-defensins (AvβD4-5, AvβD7, AvβD12), cytokines (IL-6, IL-1β, IFN-γ), and MyD88 were markedly upregulated in the reproductive tracts of SE-infected ducks (all *P* < 0.05); TLR3, TLR7, NLRC3, NLRC5, and TNF-α were significantly downregulated. These results revealed that SE infection promoted lower egg production and quality, and altered the expression of TLRs, NLRs, AvβDs, and cytokine family genes. These findings provide a basis for further investigation of the physiological and immune mechanisms of SE infection in laying ducks.

## Introduction

Food-borne salmonellosis outbreaks in humans are, in large part, caused by the consumption of contaminated poultry meat or eggs, and usually result from infection with *Salmonella enterica* serovar Enteritidis (SE) (Harker et al., 2014; Martelli & Davies, 2012). A recent epidemiological investigation revealed that *Salmonella* infection is widespread in breeding poultry in developing countries (Cha et al., 2013). Other than the chicken, the duck is a natural reservoir of SE bacteria. With increased duck production, SE is the most frequent serotype isolated from ducks in China, which has high levels of duck consumption (Gong et al., 2014). SE infection frequently results in the production of contaminated eggs, and vertical transmission and serious environmental pollution frequently occur because of infection of the reproductive organs and shedding in fecal matter.

SE causes egg contamination via internal contamination from the gut to the reproductive tract, not via contamination of the eggshell (Humphrey et al., 1991). The bacterium can colonize the vitelline membrane of unformed eggs, egg whites, or the shell membrane in the reproductive tract and cause direct contamination of egg output (Okamura et al., 2001a). In infected hens, SE is deposited in the yolk or albumen, or both, of developing eggs (Gantois et al., 2009), which suggests that egg contamination in any part of the reproductive tract is possible. A better understanding of SE infection in the various segments of the duck reproductive tract and how immune response dynamics contribute to the mechanism of SE invasion during egg formation is critical for the development of effective control measures.

SE invasion involves a number of immune factors. For example, during duck infection with SE, the higher levels of antigen-specific IgY in serum and of IgA in the gut are associated with elevated numbers of SE in the cecum (Berthelot-Herault et al., 2003). The suppression of T-lymphocyte activity is associated with infection of the reproductive tract by *Salmonella* (Johnston et al., 2012; Wigley et al., 2005). Toll-like receptors (TLRs), NOD-like receptors (NLRs), avian β-defensins (AvβDs), and many cytokines are key mediators of the immune response in various vertebrates, including avian species (Franchi et al., 2006; Ganz, 2003; Li et al., 2017; Ma et al., 2011; Ma et al., 2012; Ma et al., 2009; Zhang et al., 2017); they provide the first line of defense against potential pathogens. However, the expression patterns and functions of these immune genes vary greatly between bird species. AvβD2 is bactericidal against *Salmonella* in the chicken, but not in the turkey (Cuperus et al., 2013). The duck responds to TLR7 agonists with upregulation of mRNA encoding proinflammatory cytokines and IFN α, this upregulation elicits a response during viral infection that is not typically seen in chickens (MacDonald et al., 2008). Although growing numbers of known or putative antimicrobial defense systems have been found in the reproductive tracts of avian species (Brownlie & Allan, 2011; Ganz, 2003; Kupz et al., 2012; Ma et al., 2011; Michailidis, Theodoridis & Avdi, 2011; Soman, Arathy & Sreekumar, 2009), their expression and function in the reproductive organs of the duck have not been studied extensively.

In this study, ducks (*Anas platyrhynchos*) were infected with SE and egg production and bacterial colonization were investigated during persistent bacterial infection. In particular, we focused on the immune responses that protected from SE infection of the reproductive tract. The immune response genes expression profiles provided invaluable information for understanding the physiological and immune mechanism of SE infection in laying ducks.

## Materials and Methods

### Ethics statements

All animal experiments used in this study were approved by the Institutional Animal Care and Use Committee of Yangzhou University (Jiangsu, China) and were strictly implemented according to the regulations for experimental animals. An ordinary housing facility was used and was consistent with the national standard, Laboratory Animal Requirements of Environment and Housing Facilities (GB 14925-2001). Laboratory animal care and the animal experiment protocols and conditions conformed to the Jiangsu Administration Rule for Laboratory Animal Use.

### Bacterial strains and animals

SE (No. MY1, phage type 4) was isolated from ducks and maintained by the Key Laboratory of Animal Disease and Human Health of Sichuan Province (Deng et al., 2008). The serotypes and phage types of the bacterial isolates were determined by the National Center for Medical Culture Collection (Beijing, China). Healthy, *Salmonella*-free, 12-week-old Shaoxing ducks were obtained from the National Waterfowl Conservation Farm (Taizhou, Jiangsu, China) and were housed in isolators until use. The control group and the experimental group were kept in cages in separate rooms under identical conditions. Each duck was housed separately in a cage, provided with free access to feed and drinking water and given a shower spray in the morning and in the evening.

### Experimental infection of animals

The dose of bacteria used for the challenges was determined by pilot experiments. Briefly, 50 12-week-old Shaoxing ducks were divided into five groups with 10 ducks in each group. The respective groups were given (oral route) 2 mL SE MY1 (phosphate-buffered saline [PBS]-suspended bacteria) of 10^8^ colony forming units (CFU)/ml, 10^9^ CFU/mL, 10^10^ CFU/mL, 10^11^ CFU/mL, or 10^12^ CFU/mL every 3 days for 2 weeks. Antibody titers were detected using ELISA. The lowest challenge dose (10^11^ CFU/mL) that produced SE antibody 100% of serum was used as the inoculation dose. Our infection model was based on the results of previous studies (Deng et al., 2008). Briefly, the 12-week-old female ducks were divided into two groups. The experimental group (*n* = 130) was orally gavaged with 2 ml inoculum that contained approximately 10^11^ CFU SE. The inoculum was given every 3 days for 2 weeks*.* Birds in the non-infected control group (*n* = 20) were housed under similar environmental conditions and administered 2 ml PBS in lieu of SE. On day 14 post-infection, the IDEXX SE Ab Test was used to detect SE antibodies in the serum of all animals. Cloacal swabs were also collected every week from individuals in both groups until 13 weeks post-infection. At this point (1 day after the final cloacal swab), all birds were euthanized and the reproductive tract segments from each duck were collected, snap-frozen in liquid nitrogen, and stored at −80 °C until analysis.

### Bacteriological analysis

For enumeration of bacterial burden, cloacal swabs or homogenized tissues samples were directly inoculated onto Brilliant Green agar (BGA) (Oxoid, Basingstoke, UK) plates, which were incubated overnight at 37 °C. When negative after direct inoculation, cloacal swabs or homogenized tissue samples were incubated overnight in buffered peptone water (BPW) at 37 °C. The samples were then enriched by addition of 1 ml suspension to 9 ml brilliant green tetrathionate broth. After overnight incubation, 200 µl suspension was plated onto BGA and incubated at 37 °C for 18 h. *Salmonella* were identified on the basis of their colony morphology on BGA. The identity was confirmed by re-streaking positive samples onto ontoxylose lysine desoxycholate agar (Oxoid).

**Figure 1:**
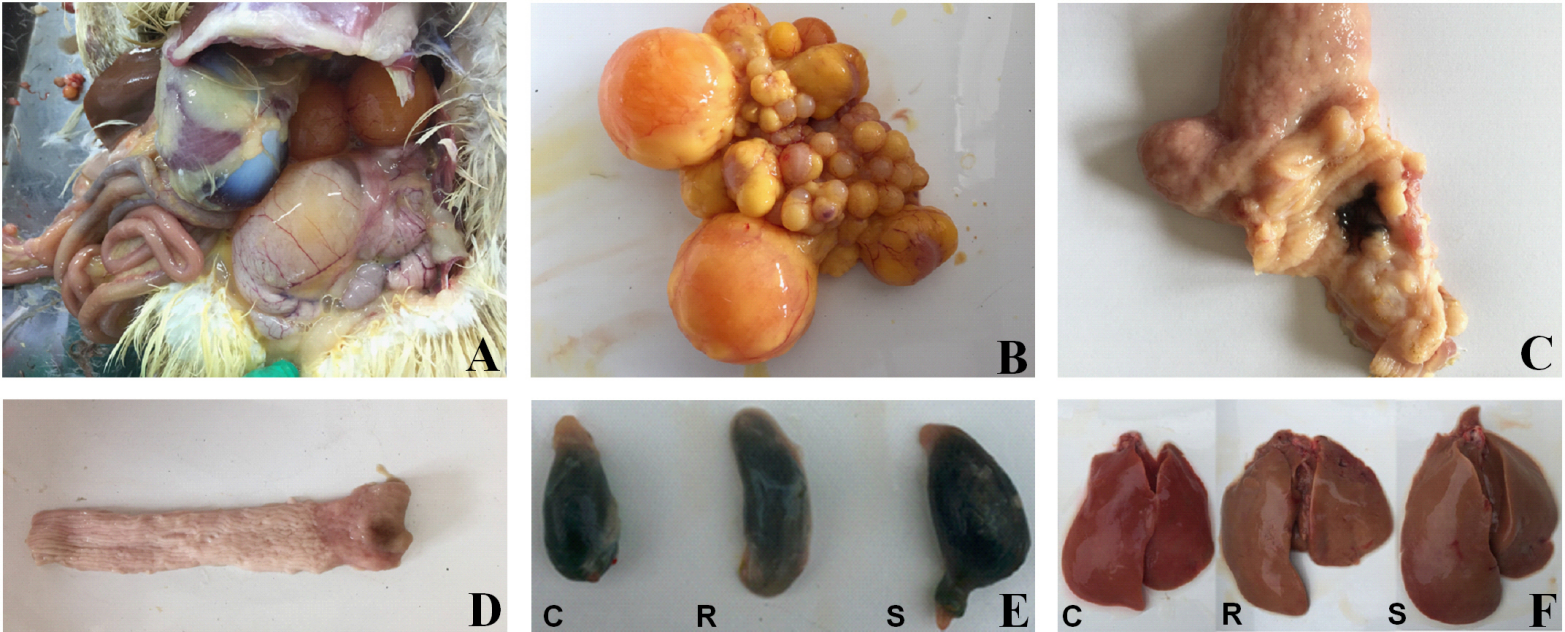
Macroscopic pathological changes in organ tissue from SE-infected ducks. In susceptible ducks, (A) the abdominal cavity contains egg yolk liquid; (B) there is evidence of ovarian inflammation, with shrunken, deformed, and disordered follicles, some of which are burst and broken; (C) there is congestion in the tubal isthmus and the mucous membrane are detached; (D) the vaginal mucosa contains black, calcified deposits; (E) the gallbladder is significantly enlarged; and (F) the livers are yellow, with bleeding from individual parts. ‘C’ indicates the control group, ‘R’ indicates the resistant group, and ‘S’ indicates the susceptible group. Photograph courtesy of Guohong Chen, July 2018.

Based on the presence of tissue lesions (Fig. 1) and on *Salmonella* isolation from SE-infected ducks, we classified infected animals as resistant or susceptible. Ten ducks from the control group and 20 ducks from the experimental group (10 resistant ducks and 10 susceptible ducks) were chosen and formed the three groups (control, resistant, susceptible) for the following experiments.

### Egg production and quality analysis

The ducks were kept in cages and the marked eggs were collected. Egg output and egg quality were monitored in the 18-week-old to 26-week-old birds. Thirty eggs were selected from the control, resistant, and susceptible groups. Egg weight, eggshell thickness, eggshell strength, egg shape index, albumen height, and yolk color were measured at 13 weeks post-infection using a Multi-function egg quality detector EMT-7300 (Robotmation, Tokyo, Japan). The data analysis of the week egg production and quality were on the individual bird basis at each day at 18, 20, 22, 24, and 26 -week-old after the individual of three groups were determinded.

### Detection of T Lymphocytes and antibody titer indices in serum

Blood from the brachial vein was collected at 13 weeks post-infection, just before euthanasia, and the serum was segregated for detection. T lymphocytes, including CD3^+^, CD4^+^, CD8^+^ cells, and antibody titers, such as IgA, IgG, and IgM, were determined using commercial ELISA kits from Nanjing Jiancheng Co., Ltd (Nanjing, China).

### SE carrier-state analysis in different segments of the duck reproductive tract

At 13 weeks post-infection, ducks were euthanized and anatomical analysis was performed. Specifically, cecal contents, large follicles, small follicles, ovarian stroma, infundibulum, magnum, uterus, isthmus, and vagina were collected, and SE colonization was detected by PCR and enumeration of numbers of CFUs. Briefly, pipetted 100 µl homogenized tissue samples were incubated overnight at 37 °C in BPW. The samples were then enriched by addition of 1 ml of this suspension to 9 ml brilliant green tetrathionate broth. After another overnight incubation, 200 µl suspension was plated onto BGA and incubated at 37 °C for 18 h. The cultures were then incubated statically at 37 °C and numbers of CFU were estimated using serial dilution (0, 10, 100, and 1,000). Each dilution was plated onto BGA (Oxoid, Basingstoke, UK).

### RNA isolation and cDNA synthesis

The total RNA was isolated from duck ovaries and oviducts using TRIzol Reagent (Life Technologies, NY, USA) and was stored at −80 °C. Reverse transcription was performed using the Takara Reverse Transcriptase M-MLV assay (RNase H-) (Takara, Dalian, China) in 10-µl reactions containing 2 µl 5X Prime Script Buffer and 8 µl RNA (<500 ng). Reaction conditions were 37 °C for 15 min, 85 °C for 5 s, followed by cooling to 4 °C. All synthesized cDNA was stored at −20 °C.

### Quantitative real-time PCR analysis

Primers for qRT-PCR specific to duck immune response genes and the SE gene, *Sdf*1 (Agron et al., 2001), are listed in Table 1. These were designed based on sequences from GenBank using Primer Premier 5.0 software. They were synthesized by Shanghai Invitrogen Biotechnology Co., Ltd. qRT-PCR was performed using SYBR Green PCR Master Mix (Takara Bio, Shiga, Japan) on an ABI PRISM 7500 HT sequence detection system (Applied Biosystems, Carlsbad, CA, USA) (*n* = 3) in 20-µl reactions containing 1 µl cDNA product, 0.6 µl, each, upstream and downstream primers, 7.8 µl ddH_2_O, and 10 µl FastStart Universal SYBR Green Master (ROX). The reaction conditions were: 50 °C for 2 min, pre-denaturation at 95 °C for 10 min; then 35 cycles of denaturation at 95 °C for 15 s, annealing at 57/59/60 °C for 30 s, and extension at 72 °C for 30 s. The data were analyzed using the 2-^ΔΔ^Ct relative quantitative method and Microsoft Excel. The expression values were normalized to the *β-actin* control. All PCR products were also subjected to 2.5% agarose gel electrophoresis and sequencing by Shanghai Sangon Biological Engineering Co., Ltd.

**Table 1:** Primers used for qRT-PCR analysis of 23 immune response genes and for detection of SE.

Primer	Primer sequence 5′-3′	Amplication size	Genebank ID
TLR1	F: 5′ TTCAGACTATATGGCAATCATCC 3′	186	JN572686.1
	R: 5′ TCATGTCTGCAAGTATCCGGTA 3′		
TLR2	F: 5′ CACTTCCGCCTATTTGACGAGA 3′	115	KX687002
	R: 5′ TTGTGTTCATTATCTTCCGCAGT 3′		
TLR3	F: 5′ AACAATTTTCCACGAATCACT 3′	138	NM_001310782
	R: 5′ CTGCAAGCTGGAATTAGCAA 3′		
TLR4	F: 5′ ATAAAAGAACTGGTCGAACCC 3′	169	NM_001310413
	R: 5′ TGCTCTCCAGAAAGTCGGTA 3′		
TLR5	F 5′ TGCCATGTATAATTCGTGCAA 3′	145	NM_001310824
	R 5′ TCCAATTTCCAAGTGCAAC 3′		
TLR7	F: 5′ GTGAATGAATGGGTGATG 3′	167	XM_013108249
	R: 5′ TGAATGCTCTGGGAAAG 3′		
TLR15	F 5′ CATCACAACCATAGCGGAGGA 3′	160	XM_005018870.3
	R 5′ CCTGAGATTTTCTTTGCCGTTT 3′		
TLR21	F 5′ ACGCGACTCCTTCCGGCTCT 3′	115	KY829021
	R 5′ GCTCACCACGCACACCGTCT 3′		
NOD1	F: 5′ GTGACTTTCTTGGGCTTATACAACA 3′	140	NM_001310381
	R: 5′ AGGCACTTCCCTCCTTCGCTA 3′		
NLRX1	F: 5′ CTGGGCTACAACTCCCTGACGGAT 3′	116	XM_021277286
	R: 5′ TTGCCCTCCTCGCTGATGTCGTT 3′		
NLRC3	F: 5′ AGCCAACCTGCTGTACGACGAA 3′	108	XM_013102028
	R: 5′ GCGTTTGCCTGAATGAAGTTCCAC 3′		
NLRC5	F: 5′ AACAGCACATTTTCACCACGTTG 3′	125	JQ868810.1
	R: 5′ TGTGTGCTCGTTCCTAAATGCAA 3′		
NLRP12	F: 5′ TTCCCATCCCAGTGAAGCGTTG 3′	129	JQ868812
	R: 5′ TTGATGTCGTGCAAATCTATTCCT 3′		
AvβD1	F: 5′ GAAACAAGGAGAAATGTCATCG 3′	183	JQ359441
	R: 5′ ATGGGGGTTGTTTCCAGGAGC 3′		
AvβD3	F: 5′ GAACTGCCACTCAGTGCAGAAT 3′	183	Ma et al. (2012)
	R: 5′ ATGGGGGTTGTTTCCAGGAGC 3′		
AvβD4	F: 5′ ATCGTGCTCCTCTTTGTGGCAGTTCA 3′	153	Ma et al. (2011)
	R: 5′ CTACAACCATCTACAGCAAGAATACT 3′		
AvβD5	F: 5′ CTCTTTGCTGTCCTCCTCCT 3′	75	JF949720.1
	R: 5′ ACAGTCCTGGGGTAATCCTC 3′		
AvβD7	F: 5′ ACCTGCTGCTGTCTGTCCTC 3′	173	JF831960
	R: 5′ TGCACAGCAAGAGCCTATTC 3′		
AvβD12	F: 5′ GGAACCTTTGTTTCGTGTTCA 3′	155	Ma et al. (2011)
	R: 5′ GAGAATGACGGGTTCAAAGC 3′		
IL-1β	F: 5′ CCGAGGAGCAGGGACTTT 3′	133	DQ393268
	R: 5′ AGGACTGTGAGCGGGTGTAG 3′		
IL-6	F: 5′ AAAGCATCTGGCAACGAC 3′	88	JQ728554
	R: 5′ GAGGAGGGATTTCTGGGT 3′		
TNF-*α*	F: 5′ GATGGGAAGGGGATGAAC 3′	144	XM_021277517
	R: 5′ ATTACAGGAAGGGCAACA 3′		
IFN-*γ*	F: 5′ ACTGAGCCAGATTGTTACCC 3′	189	AY166850.1
	R: 5′ GCCTTGCGTTGGATTTTC 3′		
β-actin	F: 5′ CCGTAAGGACCTGTACGCCAACAC 3′	208	AY251275
	R: 5′ GCTGATCCACATCTGCTGGAAGG 3′		
MyD88	F: 5′ CGCCTCGGTCTCTACCTCAACCC 3′	108	NM_001310832.1
	R: 5′ CAGAGCCTCCAGCCGCCGGAT 3′		
*Sdf*1	F: 5′ GAATCAGTATAATTCGTCAATACCTAAG 3′	293	GD165044.1
	R: 5′ ATTCAATTTCTGTCGCATATATGCTTAA 3′		

### Statistical analysis

The data analysis was performed using SPSS 17.0 software. One-way ANOVA was utilized to evaluate the significance of differences in the expression of TLRs, NLRs, AvβDs, and cytokine genes in various groups of animals. Differences between the mean values for each of the groups were estimated using Duncan’s New Multiple Range Test. A two-tailed *t*-test was performed to estimate the significance of differences between the groups of PBS and SE-infected ducks. Results were expressed as mean ± standard error of the mean (SEM) values. In all cases, differences were considered statistically significant at *P* < 0.05.

## Results

### Dynamic monitoring of long-term fecal shedding in SE-infected ducks

**Figure 2:**
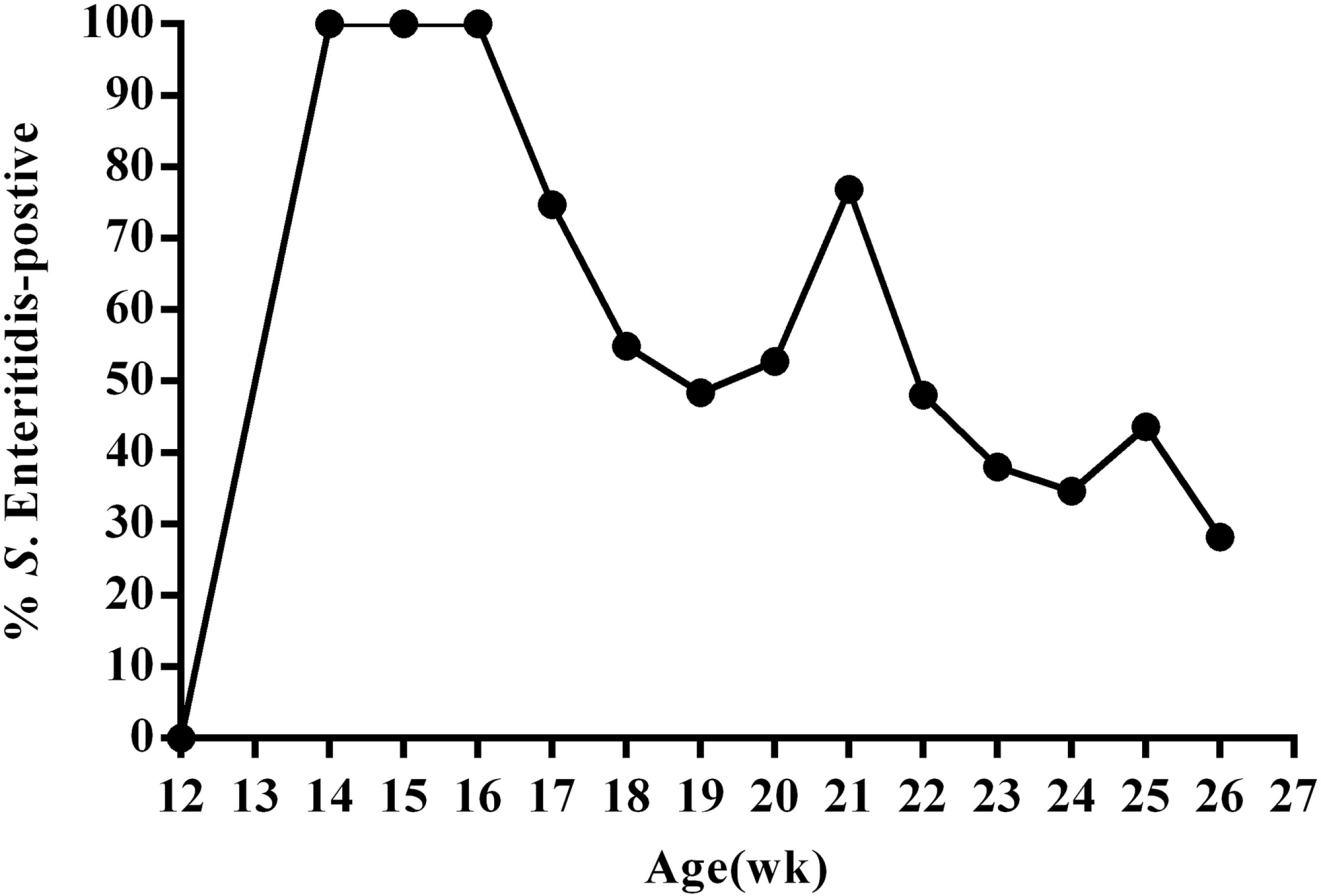
Percentage of SE-positive cloacal swabs from SE-infected ducks during the 13-week experimental period as detected by PCR and direct cultivation of microorganisms. Animals were infected with 10^11^CFU of *Salmonella enterica* serovar Enteritidis (SE) at 13 weeks post-hatch and monitored for 13 weeks post-infection.

To better understand SE infection in ducks, we orally infected 12-week-old female ducks (*n* = 130) with approximately 10^11^ CFU SE (experimental group); control animals (*n* = 20) were administered an equal volume of PBS. We then utilized the IDEXX SE Ab Test to measure SE antibodies in the serum of all animals at 14 days post-infection. The results of this test revealed that all ducks in the experimental group were infected at day 14 post-infection. We then monitored fecal shedding over time using SE-specific PCR and direct plating of samples from cloacal swabs collected every week during the 13-week study period. These data revealed that overall, the percentage of SE(+) ducks displaying fecal shedding decreased over time; although, an increasing percentage of SE(+) shedding was observed during sexual maturity (20–21 week; 24–25 week) (Fig. 2, Table S1). Most SE(+) ducks showed intermittent shedding. Some ducks had positive cloacal swab results throughout the study period. Others had negative results a few weeks post-infection and remained so for the duration of the study.

After a total of 13 weeks, all ducks were euthanized and subjected to anatomical analysis and enumeration of bacteria in tissues. Results obtained from analysis of the cecal contents generally did not correlate with the results from the last cloacal swab. However, livers from some ducks were yellow, often with portions that were bleeding; the gallbladders from these animals were significantly enlarged. We also detected signs of ovarian inflammation, with some shrunken follicles and overall deformed and disordered shape; some ovaries had burst and broken into the abdominal cavity, releasing egg yolk liquid and a foul smell. We also found congestion in the tubal isthmus, detached mucous membranes, and black, calcified deposits in the vaginal mucosa. In contrast, tissue from the resistant ducks did not contain visible lesions (Fig. 1). We defined the presence of reproductive tissue lesions and *Salmonella* isolation from SE-infected ducks as the susceptible ducks (12.8%). Ducks without detected tissue lesions and *Salmonella* isolation were the resistant (71.8%) of the oral infection ducks. *Salmonella* was isolated from the remaining 15.4% of the ducks, but there were no visible lesions (Table S2).

### SE colonization in reproductive tissues

**Figure 3:**
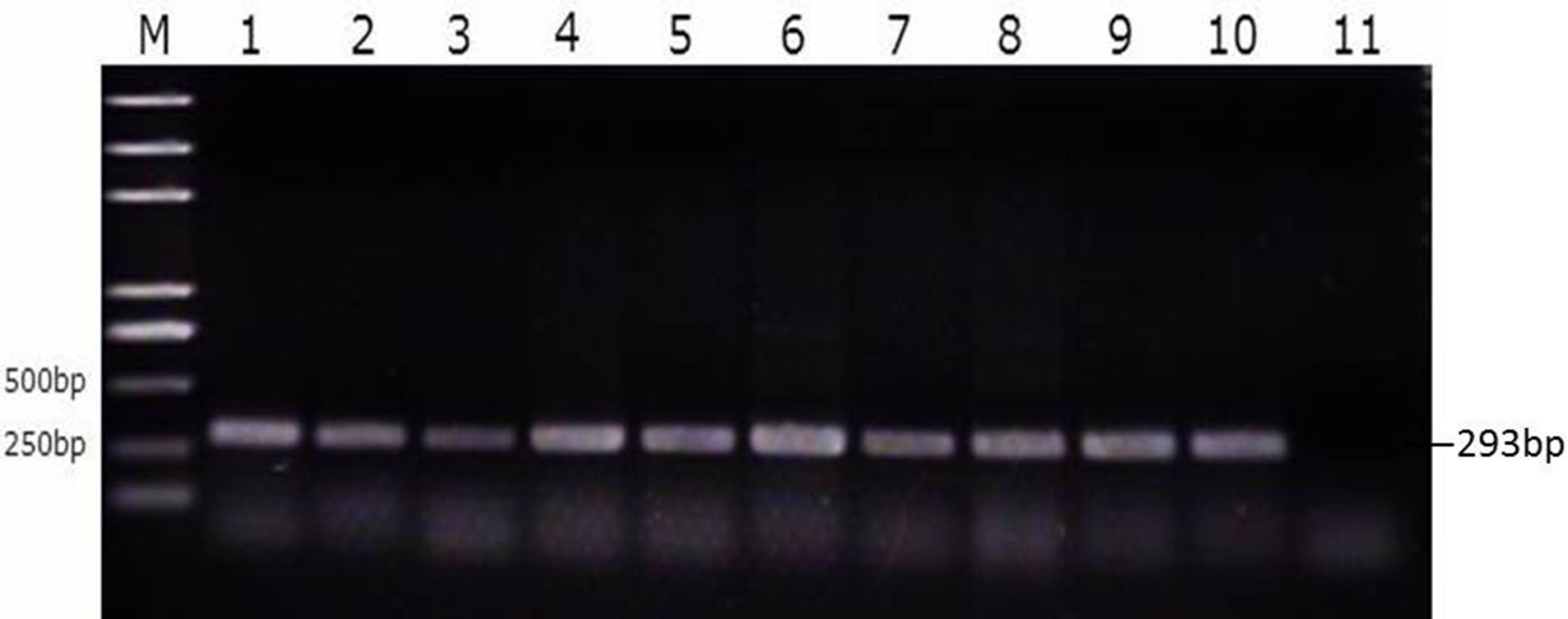
Identification of SE strains isolated from the follicle, ovarian stroma, isthmus, vagina, and cecal contents of infected ducks by PCR using Primers specific for *sdf*1. 1. Ovary stroma, 2. Small follice, 3. Large follicle, 4. Infundibulum, 5. Magnun, 6. Isthmus, 7. Uterus, 8. Vagina, 9. Cecal content, 10. Postive control, 11. Negative control.

We then divided the reproductive tracts from control group, susceptible group, and resistant group (nine from each group) animals into eight parts (large follicles, small follicles, ovarian stroma, infundibulum, magnum, uterus, isthmus, and vagina). These tissues were tested for SE as described above. That is, both the bacterial burden and colonization rate in the different segments of the reproductive tracts of the ducks were determined using PCR with primers specific for the SE *Sdf*1 gene (Fig. 3, Table S3). We found that within the duck reproductive tract, the colonization of SE was highest in vagina, moderately high in the ovarian stroma and isthmus, and lowest in the follicles (Table 2, Table S4).

**Table 2:** Bacterial burden (log CFU/g), colonization rates (%), and PCR postive rates (%) in different segments of the reproductive tract from SE-infected ducks.

Tissue	Control			Susceptible			Resistant		
	Bacterial burden (log CFU/g)	Colonization rate (%)	PCR postive rate (%)	Bacterial burden (log CFU/g)	Colonization rate (%)	PCR postive rate (%)	Bacterial burden (log CFU/g)	Colonization rate (%)	PCR postive rate (%)
Large follicles	0	0	0	+	20	20	0	0	0
Small follicles	0	0	0	3.57 ± 0.55	100	100	0	0	0
Ovary Stroma	0	0	0	5.04 ± 0.70	100	100	0	0	0
Infundibulum	0	0	0	+	20	20	0	0	0
Magnum	0	0	0	+	10	10	0	0	0
Isthmus	0	0	0	4.92 ± 0.69	100	100	0	0	0
Uterus	0	0	0	+	70	70	0	0	0
Vagina	0	0	0	5.74 ± 0.76	100	100	0	0	0

**Notes.**

“+” means that positive after enrichment.

### Effect of SE infection on egg quality and laying rate

To determine the effect of SE infection on egg production, we chose 10 ducks from the control group and 20 ducks from the experimental group (10 resistant and 10 susceptible ducks) and monitored daily egg output and egg quality. We found that animals in the susceptible group had the lowest laying rates and egg production. However, these metrics were also lower for the resistant group compared with the control animals (Table 3, Table S5). Measures of egg quality (weight, eggshell thickness, eggshell strength, albumen height, and yolk color) for eggs from the susceptible group were all lower than those of the control group (*P* < 0.05). Egg weight, eggshell strength, and albumen height for eggs from the resistant group were lower than those of the control group (*P* < 0.05) (Table 4, Table S6). Eggs from the resistant group had greater eggshell thickness and deeper yellow yolk color, compared with the susceptible group (*P* < 0.05). The percentages of eggs containing blood spots and meat spots were highest in the susceptible group; the resistant group also had higher values for these characteristics, compared with the control group. Egg shape indices were similar for all three groups.

**Table 3:** Laying rate and egg production during SE infection.

Weekly age	Laying rate (%)			Egg production		
	Control	Susceptible	Resistant	Control	Susceptible	Resistant
18w	11.43 ± 10.69^a^	0 ± 0.0^b^	0 ± 0.0^b^	8 ± 1.07^a^	0 ± 0.0^b^	0 ± 0.0^b^
20w	40 ± 11.55^a^	0 ± 0.0^b^	17.14 ± 7.56^c^	28 ± 1.15^a^	0 ± 0.0^b^	12 ± 0.76^c^
22w	74.29 ± 12.72^a^	38.57 ± 13.45^b^	60 ± 21.6^c^	52 ± 1.27^a^	27 ± 1.35^b^	42 ± 2.16^c^
24w	98.57 ± 3.78^a^	67.14 ± 13.8^b^	92.86 ± 7.56^a^	69 ± 0.38^a^	47 ± 1.38^b^	65 ± 0.76^a^
26W	100 ± 0.0^a^	87.14 ± 7.56^b^	97.14 ± 4.88^a^	70 ± 0.0^a^	61 ± 0.76^b^	68 ± 0.49^a^

**Notes.**

In the same horizontal, values with different lowercases mean significant difference (*P* < 0.05), values with the same letter or without letter mean no significant difference (*P* > 0.05).

**Table 4:** Assessment of egg quality during SE infection.

Items	Control	Susceptible	Resistant
Egg weight, g	63.10 ± 2.97^a^	60.26 ± 2.78^b^	60.29 ± 2.93^b^
Eggshell thickness, mm	0.333 ± 0.031^a^	0.308 ± 0.033^b^	0.336 ± 0.029^a^
Eggshell strength, kg/cm^2^	4.65 ± 0.36^a^	4.22 ± 0.60^b^	4.27 ± 0.46^b^
Egg shape index	1.34 ± 0.06	1.33 ± 0.08	1.35 ± 0.05
Albumen height, mm	6.40 ± 0.49^a^	5.72 ± 0.52^b^	5.76 ± 0.65^b^
Yolk color	7.44 ± 0.52^a^	7.83 ± 0.63^b^	7.20 ± 0.45^a^
Blood spot ratio, %	0	46.67	23.33
Meat spot ratio, %	0	40	10
SE(+), %	0	23.33	0

**Notes.**

In the same horizontal, values with different lowercases mean significant difference (*P* < 0.05), values with the same letter or without letter mean no significant difference (*P* > 0.05).

### Effect of SE infection on T lymphocytes and antibody titers in serum

To further determine the effect of SE infection on immune responses, we measured T lymphocytes and serum antibody titers at 13 weeks post-infection in SE-infected and control animals; all results are summarized in Table 5 (Table S7). We found that the levels of CD3^+^, CD4^+^, and CD4^+^/CD8^+^ T cells in the susceptible group were lower than in the control group or the resistant group (*P* < 0.05). Levels of CD8^+^T cells, IgA, IgG, and IgM were higher in the susceptible group than in either the control or the resistant group (*P* < 0.05). We detected greater numbers of CD3^+^, CD4^+^, and CD4^+^/CD8^+^ T cells and increased IgA, IgG, and IgM, in the resistant group compared with the susceptible group (*P* < 0.05). However, the resistant animals had fewer CD8^+^ T cells than the susceptible animals (*P* < 0.05).

**Figure 4:**
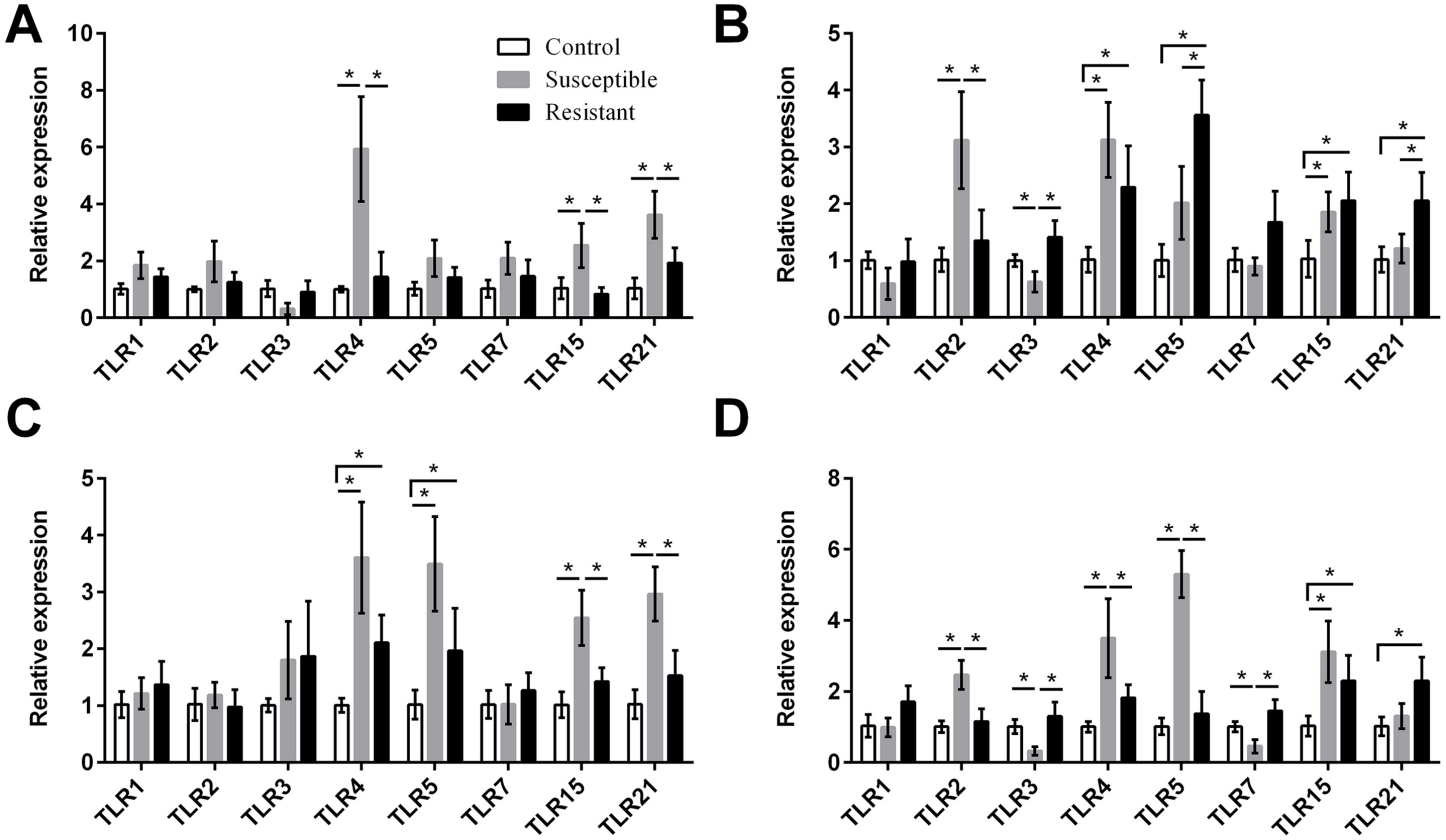
Changes in the expression of TLR mRNA in four different segments of the duck reproductive tract following infection with SE MY_1_. (A) Follicle; (B) ovarian stroma; (C) isthmus; (D) vagina. Values shown (fold change) are the mean ± SEM of three independent experiments. Note: * indicates that the difference in the expression levels between two groups of animals are significantly different (*P* < 0.05).

**Table 5:** Measurement of T lymphocyte populations and serum antibody titers during SE infection.

Items	Control	Susceptible	Resistant
CD3^+^ (ng/µl)	368.42 ± 57.93^a^	277.99 ± 49.70^b^	325.13 ± 73.48^a^
CD4^+^ (ng/µl)	289.01 ± 35.05^a^	246.34 ± 23.96^b^	306.97 ± 34.19^a^
CD8^+^ (ng/µl)	239.23 ± 27.76^a^	288.02 ± 20.24^b^	224.09 ± 25.03^a^
CD4^+^/CD8^+^	1.22 ± 0.22^a^	0.86 ± 0.12^b^	1.40 ± 0.28^a^
IgA (µg/ml)	289.93 ± 40.32^a^	793.95 ± 92.28^b^	352.72 ± 86.06^a^
IgG (µg/ml)	761.99 ± 67.48^a^	1590.64 ± 175.84^b^	825.12 ± 54.35^a^
IgM (µg/ml)	310.15 ± 36.19^a^	519.22 ± 41.08^b^	352.18 ± 44.63^a^

**Notes.**

In the same vertical, values with different lowercases mean significant difference (*P* < 0.05), values with the same letter or without letter mean no significant difference (*P* > 0.05).

### Expression of immune response genes in the follicle, ovarian stroma, isthmus, and vagina of SE-infected ducks

We next focused on the additional immune factors TLRs, NLRs, AvβDs, and cytokines, and investigated the expression of these four families of proteins in the follicle, ovary stroma, isthmus, and vagina during SE infection. For the TLR family, we found that expression of *TLR2*, *TLR4*, *TLR5*, *TLR15*, and *TLR21* mRNA was upregulated in the reproductive tract after SE challenge (*P* < 0.05), and that the various tissues showed different trends in expression (Fig. 4, Table S8). In the susceptible ducks, expression of *TLR2* and *TLR4* was increased in the ovary stroma and vagina, respectively, compared with resistant ducks. Conversely, expression of *TLR5* and *TLR21* in the ovarian stroma was significantly upregulated in the resistant animals, compared with the susceptible animals. In the resistant ducks, we detected significantly higher levels of *TLR3* in the ovarian stroma and vagina and of *TLR7* in the vagina, compared with the susceptible ducks.

**Figure 5:**
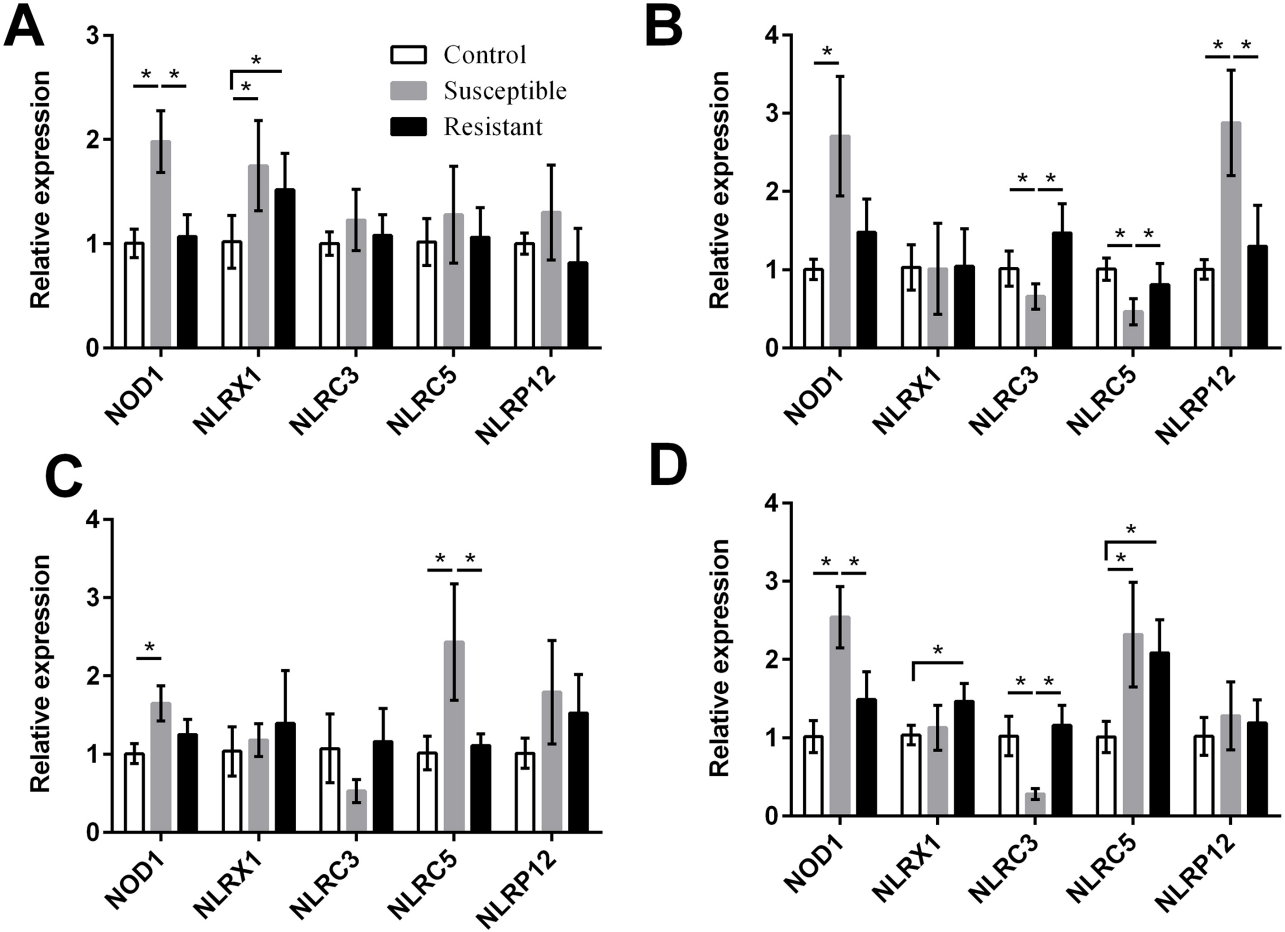
Changes in the expression of NLR mRNA in four different segments of the duck reproductive tract following infection with SE MY_1_. (A) follicle; (B) ovarian stroma; (C) isthmus; (D) vagina. Values shown (fold change) are the mean ± SEM of three independent experiments. Note: * indicates that the difference in the expression levels between two groups of animals are significantly different (*P* < 0.05).

For the NLRs (NOD1, NLRX1, NLRC3, NLRC5, and NLRP12) we found that the *NOD1* was upregulated after SE challenge (*P* < 0.05), as were *NLRX1* in the follicle, *NLRP12* in the stroma, and *NLRC5* in the isthmus and vagina (*P* < 0.05) (Fig. 5, Table S8). Levels of *NOD1*, *NLRC3*, *NLRC5,* and *NLRP12* mRNA were significantly different between the susceptible ducks and resistant ducks. We found that in susceptible ducks, expression of *NOD1* in the follicle and vagina and of *NLRP12* in the ovarian stroma was significantly higher than in these tissues in resistant ducks. In contrast, *NLRC3* and *NLRC5* levels were lower in the stroma and vagina of susceptible animals compared with resistant animals (*P* < 0.05).

**Figure 6:**
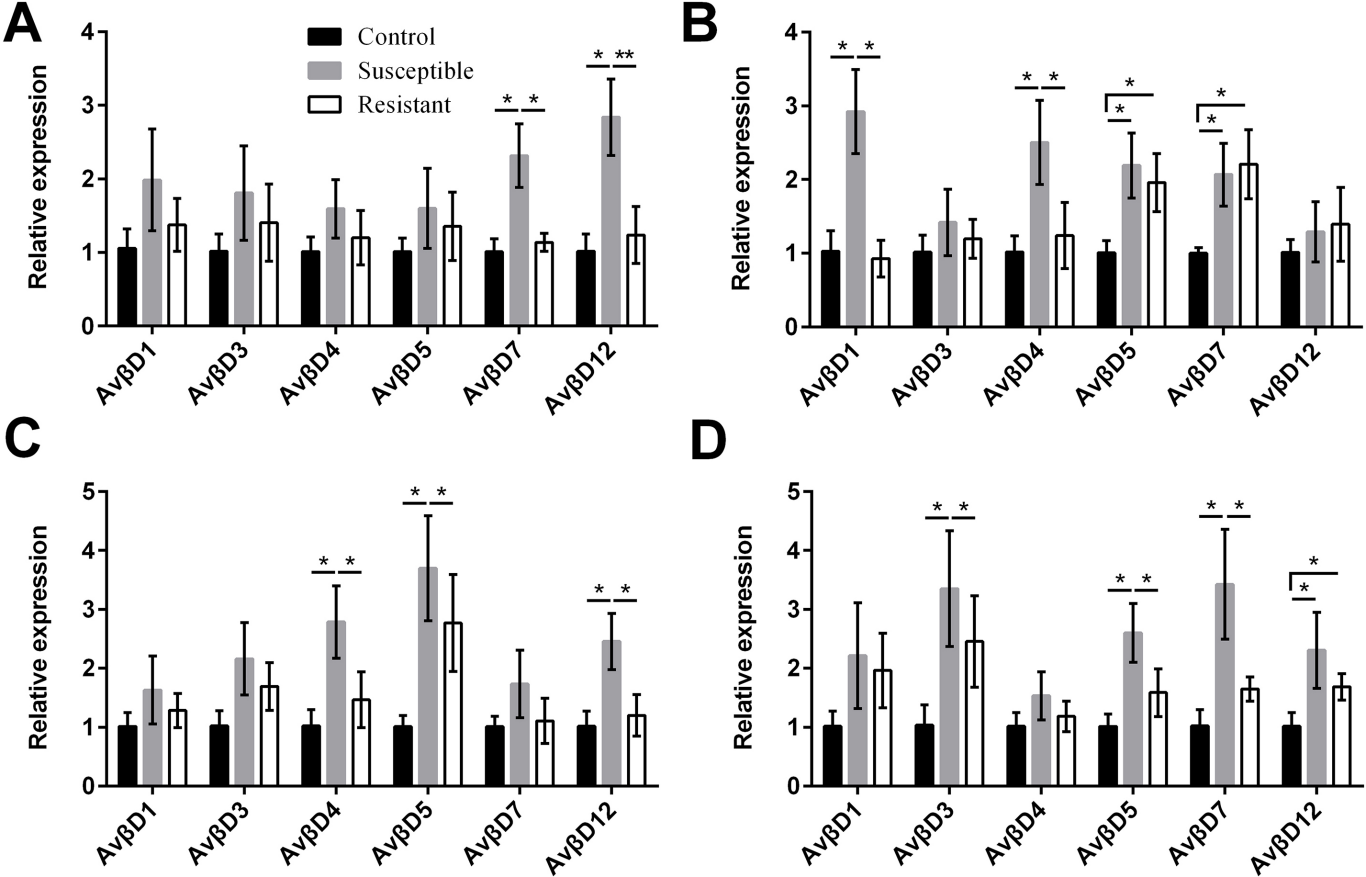
Changes in the expression of AvβD mRNA in four different segments of the duck reproductive tract following infection with SE MY_1_. (A) Follicle; (B) ovarian stroma; (C) isthmus; (D) vagina. Values shown (fold change) are the mean ± SEM of three independent experiments. Note: * indicates that the difference in the expression levels between two groups of animals are significantly different (*P* < 0.05).

We performed an analysis of AvβD expression profiles in SE-infected ducks and controls. The results suggested that the Av βD proteins had diverse roles in immune regulation in response to SE infection. In general, we measured increased expression of AvβD3-5, AvβD7, and AvβD12 in SE-infected vs. control ducks (*P* < 0.05) (Fig. 6, Table S8). In susceptible ducks, we detected elevated expression of AvβD7 and AvβD12 in follicles, AvβD1 and AvβD4 in the stroma, AvβD4-5 and AvβD12 in the isthmus, and AvβD3, AvβD5, and AvβD7 in the vagina, compared with the resistant group (*P* < 0.05). Compared with the TLR and NLR family members, expression of different AvβD family genes showed more tissue specificity after infection.

**Figure 7:**
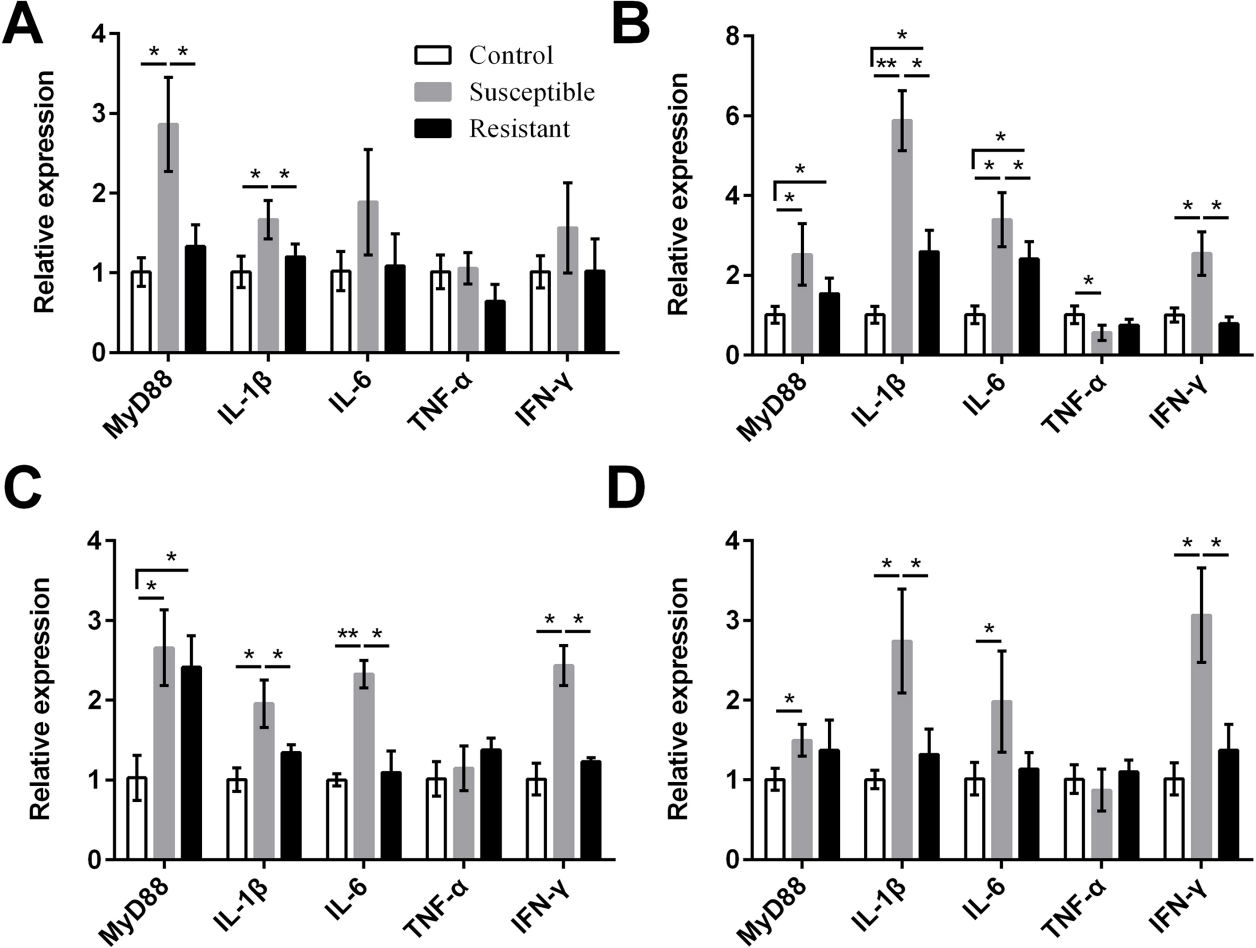
Changes in the expression of cytokine mRNA in four different segments of the duck reproductive tract following infection with SE MY_1_. (A) Follicle; (B) stroma; (C) isthmus; (D) vagina. Values shown (fold change) are the mean ± SEM of three independent experiments. Note: * indicates that the difference in the expression levels between two groups of animals are significantly different (*P* < 0.05).

We also measured expression of various proinflammatory cytokines and found that MyD88, IL-1β, IL-6, and IFN-γ were upregulated in the reproductive tract of the SE-infected ducks, compared with the control ducks (Fig. 7, Table S8). Expression of most of these cytokines in the susceptible group was significantly higher than in the resistant group. Conversely, we detected reduced expression of TNF-α in the ovarian stroma of susceptible ducks, compared with control animals (*P* < 0.05).

## Discussion

*Salmonella* infection is prevalent in breeding poultry, including chickens and ducks. However, most studies of SE-infected poultry have focused on the processes occurring within the 13-week post-infection period (De Vylder et al., 2009; Gast et al., 2015; Van Immerseel et al., 2004). Thus, analysis of recessive infection in long-term research studies is rare. In this study, ducks were infected with SE and were systematically monitored for fecal shedding over a 13-week period. We found persistent SE infection and intermittent long-term shedding, and lower laying rates and egg quality. These results are consistent with the typical characteristics of *Salmonella* infection in poultry (Shivaprasad et al., 1990). Generally, during SE infection, the bacterium enters the esophagus, and then invades the intestinal epithelial cells via macrophages, which spread throughout the avian body system (Okamura et al., 2001a). In our study, we found that some ducks’ ovarian tissues were invaded by SE following a route of entry via the digestive tract; SE colonized the ovarian stroma, isthmus, small follicle, and vagina. In chickens, different infection models have also revealed that SE colonizes the tubular glands of the isthmus in the oviduct, which leads to contamination of the shell membrane (Miyamoto et al., 1997; Okamura et al., 2001a; Okamura et al., 2001b). Howard et al. (2005) found that the immature white follicles are more susceptible to *Salmonella* invasion than the relatively mature larger yellow follicles, and that a large number of SE infections occur in the vagina. Gast & Holt (2001) further elucidated the process by which *Salmonella* colonizes the ovary. It forms deposits on the stroma outside the vitelline membrane, rather than in the nutritious egg yolk. Taken together, these results indicate that SE has preferred areas of ovarian colonization. We did not detect SE colonies in the ovarian tissue of SE-resistant ducks, which indicated that SE ducks had greater immune competence and cleared the SE during long-term asymptomatic infection. We also found that long-term asymptomatic infection with SE resulted not only in decreased egg production and egg quality, it also caused egg contamination. The effectiveness of the strategies used for prevention and control of *Salmonella* infections during production should be improved to avoid losses.

The antibody response was assessed in addition to the bacterial distribution in the reproductive tract, laying rate, and egg quality. T-lymphocyte subpopulations were detected during the SE challenge. The results indicated that T lymphocytes decreased sharply in SE-infected susceptible ducks, particularly the CD4^+^ and CD4^+^/CD8^+^ T cells. This change was consistent with the loss of ability to control disease at 13 weeks post-infection. These changes were likely to increase the susceptibility of the ovarian and oviductal epithelium to infection. Hens at point-of-lay have increased susceptibility for infection with *Salmonella* (Johnston et al., 2012). However, we found that IgA, IgG, and IgM increased in the susceptible ducks after 13 weeks post-infection. This change was related to the concentrations of SE in the reproductive tissues. This inconsistency may be because individual serum antibody titer levels are not related to SE susceptibility; humoral responses are linked to high bacterial colonization rates and severe systemic infection (Beal & Smith, 2007; Berthelot-Herault et al., 2003).

SE was recognized by pattern recognition receptors (PRRs) of the innate immune system. PRRs, most notably the TLRs and NLRs, are the first component of the immune system to detect host invasion by pathogens, initiating immune responses and forming the crucial link between innate and adaptive immunity (Kawai & Akira, 2010; Schroder & Tschopp, 2010). In our study, the expression profiles of immune responsive genes were examined in the reproductive tract organs of infected and control ducks. We found that SE infection promoted the largest increase in expression of TLR2, TLR4-5, TLR15, TLR21, NOD1, NLRX1, NLRP12, AvβD3-5, AvβD7, and AvβD12, as well as MyD88, IL-1β, IL-6, and IFN-γ, in the duck reproductive tract. Our study revealed reduced expression of TLR3 in the ovarian stroma and vagina and of TLR7 in the vagina of the resistant ducks. Given that TLR3 and TLR7 are antiviral receptors (Iqbal, Philbin & Smith, 2005) that are induced in response to viral challenge, it is not surprising that bacterial infection did not enhance their expression. For the NODs, we detected an upregulation of *NLRP12* transcript in the ovarian stroma of susceptible compared with resistant ducks. NLRP12 was originally described for its ability to induce NF-κB and to promote caspase-1 activation. In contrast, NOD1 and NLRX1, which are elevated in the follicle of susceptible vs. resistant ducks, are likely to promote host recognition of SE. Different Av βD genes show tissue specificity after SE infection because AvβD expression is affected by factors including species, tissue, gender, and age (Yoshimura, 2015; Yoshimura et al., 2006). *TNF-* α** was downregulated in the ovarian stroma in the resistant compared with the control ducks. Because cells undergo apoptosis or immune suppression to maintain the homeostasis of ovarian stroma, this result might have been due to the numerous bacteria colonizing the ovarian stroma and causing persistent inflammation that led to immune inhibition (Woods, Schorey & Johnson, 2009). Study results have suggested that the innate immune system of the ovary and oviduct has been highly developed to recognize components of microbes. This system may have a crucial role in the local defense against infection of the reproductive tract by pathogenic microbes. Hence, we found that compared with the follicle, fluctuations in expression of TLRs, NLRs, AvβDs, and cytokines were more pronounced in the ovarian stroma, isthmus, and vagina, possibly because the egg yolk liquid contains components with inherent immune protection capabilities.

## Conclusions

In conclusion, our results indicated that SE is associated with long-term colonization of the duck reproductive tract that results in lower egg production and quality. The differences in expression of TLRs, NLRs, AvβDs, and cytokine family genes induced in susceptible and resistant ducks infected with SE offer new insights into the physiological and immune mechanisms of SE infection in laying ducks.

## Additional Information and Declarations

### Data Availability

The following information was supplied regarding data availability: Chen, Guohong (2019): Effect of *Salmonella enterica* serovar Enteritidis infection on egg production and immune response of the laying duck. figshare. Fileset. https://doi.org/10.6084/m9.figshare.6916271.v1.
